# High-dose fenoldopam reduces postoperative neutrophil gelatinase-associated lipocaline and cystatin C levels in pediatric cardiac surgery

**DOI:** 10.1186/cc10295

**Published:** 2011-06-29

**Authors:** Zaccaria Ricci, Rosa Luciano, Isabella Favia, Cristiana Garisto, Maurizio Muraca, Stefano Morelli, Luca Di Chiara, Paola Cogo, Sergio Picardo

**Affiliations:** 1 Pediatric Cardiac Anesthesia/Intensive Care Unit, Department of Pediatric Cardiology and Cardiac Surgery, Bambino Gesù Children's Hospital, Piazza S. Onofrio 4, 00165, Rome, Italy; 2 Clinical Laboratory, Department of Clinical Medicine, Bambino Gesù Children's Hospital, Piazza S. Onofrio 4, 00165, Rome, Italy; 3 Emergency Department Unit, Bambino Gesù Children's Hospital, Piazza S. Onofrio 4, 00165, Rome, Italy

## Abstract

**Introduction:**

The aim of the study was to evaluate the effects of high-dose fenoldopam, a selective dopamine-1 receptor, on renal function and organ perfusion during cardiopulmonary bypass (CPB) in infants with congenital heart disease (CHD).

**Methods:**

A prospective single-center randomized double-blind controlled trial was conducted in a pediatric cardiac surgery department. We randomized infants younger than 1 year with CHD and biventricular anatomy (with exclusion of isolated ventricular and atrial septal defect) to receive blindly a continuous infusion of fenoldopam at 1 μg/kg/min or placebo during CPB. Perioperative urinary and plasma levels of neutrophil gelatinase-associated lipocaline (NGAL), cystatin C (CysC), and creatinine were measured to assess renal injury after CPB.

**Results:**

We enrolled 80 patients: 40 received fenoldopam (group F) during CPB, and 40 received placebo (group P). A significant increase of urinary NGAL and CysC levels from baseline to intensive care unit (ICU) admission followed by restoration of normal values after 12 hours was observed in both groups. However, urinary NGAL and CysC values were significantly reduced at the end of surgery and 12 hours after ICU admission (uNGAL only) in group F compared with group P (*P *= 0.025 and 0.039, respectively). Plasma NGAL and CysC tended to increase from baseline to ICU admission in both groups, but they were not significantly different between the two groups. No differences were observed on urinary and plasma creatinine levels and on urine output between the two groups. Acute kidney injury (AKI) incidence in the postoperative period, as indicated by pRIFLE classification (pediatric score indicating Risk, Injury, Failure, Loss of function, and End-stage kidney disease level of renal damage) was 50% in group F and 72% in group P (*P *= 0.08; odds ratio (OR), 0.38; 95% confidence interval (CI), 0.14 to 1.02). A significant reduction in diuretics (furosemide) and vasodilators (phentolamine) administration was observed in group F (*P *= 0.0085; OR, 0.22; 95% CI, 0.07 to 0.7).

**Conclusions:**

The treatment with high-dose fenoldopam during CPB in pediatric patients undergoing cardiac surgery for CHD with biventricular anatomy significantly decreased urinary levels of NGAL and CysC and reduced the use of diuretics and vasodilators during CPB.

**Trial registration:**

Clinical Trial.Gov NCT00982527.

## Introduction

Cardiopulmonary bypass (CPB) represents a unique clinical circumstance in which nearly all aspects of perfusion can be determined by clinicians. So far, considerable controversy relates to appropriate management of physiologic variables during pediatric CPB (perfusion pressure, bypass flow rates, type of flow (pulsatile versus nonpulsatile), hematocrit values, systemic oxygen delivery (DO_2_), temperature, and acid-base management), resulting in significant differences in how bypass is conducted in cardiac centers [1,2]. In light of this, pathogenesis of CPB-associated renal dysfunction has not been fully elucidated [3,4], and no proven effective prophylaxis or treatment has been established. Acute kidney injury (AKI) after cardiac operations with CPB is a life-threatening complication, with a reported incidence of up to 36% [5]. When dialytic treatment is required, the mortality rate may reach 50% [6,7]. Various factors related to the CPB procedure have been advocated as possible determinants of AKI. They include CPB duration, red blood cell fragmentation and hemolysis, sublethal red cell damage, resulting in altered rheologic properties, a low perfusion pressure, low pump flow, severe hemodilution, and low DO_2_[8-10]. In children, after CPB, the combined effects of hypothermia, nonpulsatile perfusion, and reduced mean arterial pressure are involved in the release of angiotensin, renin, catecholamines, and antidiuretic hormone. These circulating substances promote renal vasoconstriction and reduce renal blood flow [11]. Furthermore, the glomerular filtration rate, creatinine clearance, and medullary concentrating capacity are substantially reduced in neonates and young infants, with a net result of increased total body water, increased organ weight (for example, lungs, heart), and greater difficulty with postoperative weaning from ventilatory support [11].

Creatinine, urea nitrogen levels, or urine output are only very late markers for the diagnosis of AKI [12,13]. Neutrophil gelatinase-associated lipocalin (NGAL) and cystatin C (CysC) have been recently suggested to be early AKI biomarkers in both adult and pediatric cardiac surgery [14-17]. Different pharmacologic approaches have been experimented with to optimize organ perfusion during CPB. In particular, phentolamine [18], phenoxybenzamine [19], and sodium nitroprusside [20] have been used, because of their vasodilative properties, to manage hypoxic vasoconstriction on CPB, with conflicting results. These agents have rarely been tested with clinical biomarkers of kidney function [21]; typically, diuretics have been the mainstay of promoting renal function and urine flow after pediatric CPB [22,23]. Fenoldopam mesylate is a short-acting dopamine-1 (DA-1) agonist with antihypertensive and vasodilative properties [24] that has been used during adult cardiac surgery with conflicting results [25,26]. It appears to improve renal function in clinical situations of reduced blood flow by increasing renal blood flow to both the cortex and medullary regions [24]. In pediatric critically ill patients and pediatric cardiac surgery, fenoldopam has been proposed as a nephroprotective agent with additional diuretic properties [27,28]. In light of this, fenoldopam would appear to be the optimal drug during pediatric CPB, to optimize organ perfusion by systemic vasodilation with a potential specific benefit on renal function. Its commonly used clinical dosage ranges from 0.1 to 0.3 μg/kg/min [29,30]. Recently, this agent has been evaluated for controlled hypotension during pediatric abdominal surgery, and the dosage range has been reported effective between 0.8 and 1.2 μg/kg/min. Doses greater than 1.2 μg/kg/min resulted in increasing heart rate without additional reduction in blood pressure [31].

The present study aimed to evaluate the effects of high-dose fenoldopam infusion (1 μg/kg/min) on protecting the kidney from CPB-associated AKI, by perioperative measurements of urinary and plasma NGAL and CysC levels in infants with CHD.

## Materials and methods

### Study design

A single-center prospective double-blind randomized placebo-controlled trial was performed at a tertiary pediatric hospital. The study was approved by the local Ethics Committee ("Comitato Etico per la Sperimentazione Clinica"; approval number, 250) and was registered in the Protocol Registration System (Clinical Trial. Gov Id: NCT00982527).

### Objectives

The primary end point of this study was to assess whether high-dose fenoldopam infusion during CPB affects renal function by perioperative urinary and plasma NGAL, CysC, and Crea levels measurement. Secondary outcomes were the evaluation of postoperative AKI incidence, as indicated by pRIFLE (pediatric score indicating Risk, Injury, Failure, Loss of function, and End-stage kidney disease level of renal damage) classification [32]; high-dose fenoldopam infusion efficacy was assessed by CPB perfusion features in the two groups (see later) and by the requirement of diuretics and vasodilators during the CPB period. Finally, the number of times that the vasodilator infusion was halted because of hypotension (or other side effects) was recorded as a measure of fenoldopam safety.

### Study population

Inclusion criteria were age younger than 1 year, need for cardiac surgery, and CPB for correction of CHD with biventricular anatomy. Exclusion criteria were surgery for simple ventricular septal defect and atrial septal defect, the presence of preoperative renal dysfunction, predetermined need for deep hypothermic circulatory arrest (DHCA), urgent procedures, and the absence of informed consent. The study was conducted between July 2009 and July 2010.

### Randomization procedure

The allocation sequence was generated by using two computerized random-generation programs stratified by two age groups: (a) neonates (surgery before 30 days after birth) and (b) infants (surgery between 31 and 365 days after birth). To recruit the same proportion of cases, the number of enrolled neonates was limited to 13 per group (32%), reflecting the institutional rate of neonatal surgery. The patients were evaluated for eligibility the day before surgery, and informed consent was obtained from both parents. After recruitment, the day of operation, sealed envelopes containing the allocation group were opened by a nurse who did not participate in the study or in data collection. The same nurse was in charge of preparing the infusions that were blindly given to the perfusionist. The rest of the nurses and medical staff in the operation room and in the intensive care unit were blinded to group assignment.

### Interventions

All patients received general anesthesia with sevorane inhalation at induction and then midazolam (0.05 mg/kg/h), fentanyl (5 μg/kg/h), and cisatracurium (0.08 mg/kg/h) infusion for maintenance. CPB was always conducted with open circuits, alpha stat strategy, and blood cardioplegia, targeting CPB flow to 150 ml/kg/min, hematocrit to 35%, and moderate hypothermia with the temperature ranging between 25°C and 32°C. During CPB, boluses of phentolamine (0.1 μg/kg) were administered when perfusion pressure at full perfusion flow increased over 50 mm Hg. Patients were blindly allocated to the study group (group F) or to placebo (group P) after anesthesia induction. A continuous infusion of fenoldopam at the dose of 1 μg/kg/min was administered from CPB start to CPB weaning. The drug was always prepared in 25 ml of saline and infused at a rate of 1 ml/h. Group P received a saline solution, infused at the same rate as that of fenoldopam. Infusion was administered directly into the CPB reservoir. No changes to the intraoperative standard of care were applied during the study period: in particular, fluid balance was controlled by conventional ultrafiltration before CPB weaning (about 50 ml/kg of ultrafiltered volume) and by furosemide administration (1 mg/kg) if the urine output before CPB weaning was less than 1 ml/kg/h. The attending anesthesiologist was authorized to interrupt drug infusion due to suspicion of side effects such as excessive hypotension, arrhythmias, or intolerance.

### Data collection

Demographic and baseline characteristics of included patients were collected after enrolment. Urinary output, plasma and urinary levels of creatinine (respectively, pCrea and uCrea), NGAL (respectively, pNGAL and uNGAL), and CysC (respectively, pCysC and uCysC), blood lactates concentration, superior vena cava oxygen saturation (ScvO_2_) and cerebral near-infrared spectroscopy (rSO_2_c) value were measured before surgery (t0), at the end of the surgical procedure (t1), and 12 hours after ICU admission (t2). Creatinine levels and urinary output were recorded in the first 96 postoperative days to compute pRIFLE classification.

PCrea and uCrea levels were directly sent to the laboratory and measured with the Jaffe assay (ADVIA Chemistry Systems, Siemens, Munich, Germany). Urine samples for uNGAL and uCysC were collected in dedicated sterile vials, centrifuged at 2,000 rpm, and the supernatant immediately stored at -80°C. Blood samples for pNGAL and pCysC were collected in sterile vials containing heparin as an anticoagulant, centrifuged at 3,600 rpm, and the supernatant immediately stored at -80°C. Plasma and urinary NGAL were measured with a sandwich enzyme-linked immunosorbent assay (R&D Systems, Minneapolis, MN, U.S.A.), whereas plasma and urinary cystatin C were evaluated with nephelometry (BN ProSpec; Siemens).

All CPB data (pump flow, minimum temperature, hematocrit, perfusion pressure, furosemide boluses, phentolamine boluses, hemofiltration volume) were prospectively collected by perfusionists, and the average values per each CPB session of every patient were recorded. Average indexed DO_2 _and systemic vascular resistances (SVRs) were calculated for each patient during CPB. DO_2 _was calculated as follows: CPB pump flow × arterial oxygen content (assumed as hemoglobin concentration × hemoglobin saturation × 1.34 + 0.003 × arterial oxygen tension). SVRs were calculated as follows: 80 × (CPB pressure-central venous pressure)/CPB pump flow. At ICU admission and after 24 hours, in the absence of other direct instrumental measures of cardiac performance, the hemodynamics was evaluated with the mean arterial pressure (MAP) and inotropic score (IS) that indicates different inotropic and vasopressor drug regimens, reflecting dosage of vasoconstrictors/inotropic drugs. IS was calculated as [33]: dopamine μg/kg/min × 1 + dobutamine μg/kg/min × 1 + milrinone μg/kg/min × 15 + epinephrine μg/kg/min × 100. Survival, length of mechanical ventilation, and length of ICU stay also were recorded. Risk-adjusted classification for congenital heart surgery (RACHS-I) was used to compare the severity of surgical risk in the populations [34]. All data were recorded on an Access-based database specifically prepared for this study.

### Statistical analysis

Intention to treat (ITT) was applied, and all enrolled patients were considered for statistical analysis at the end of the study. The χ^2 ^test was used to compare categoric variables. To compare continuous variables, a Mann-Whitney test or Student *t *test was used, according to the variable distribution. Two-way analysis of variance was used to compare continuous variables over time between the two groups, with the Bonferroni *post hoc *test for each time point. All data are presented as mean and standard deviation (SD). A *P *value < 0.05 was considered significant. Statistical analysis was performed with the GraphPad Prism 5.0 software package (GraphPad Software, San Diego, CA, U.S.A.).

The study was powered on the primary outcome based on a previous landmark study [15]: considering a mean (SD) of uNGAL level, 2 hours after CPB weaning, of 150 (90) ng/ml, and a 50 ng/ml uNGAL difference between the two groups, to achieve an 80% statistical power with an alpha error of 0.05; the number of patients was calculated to be 40 for each group. An interim analysis was planned after enrolment of 40 patients, and a stopping rule was determined in case of major morbidity detected in this phase.

## Results

**Figure 1:**
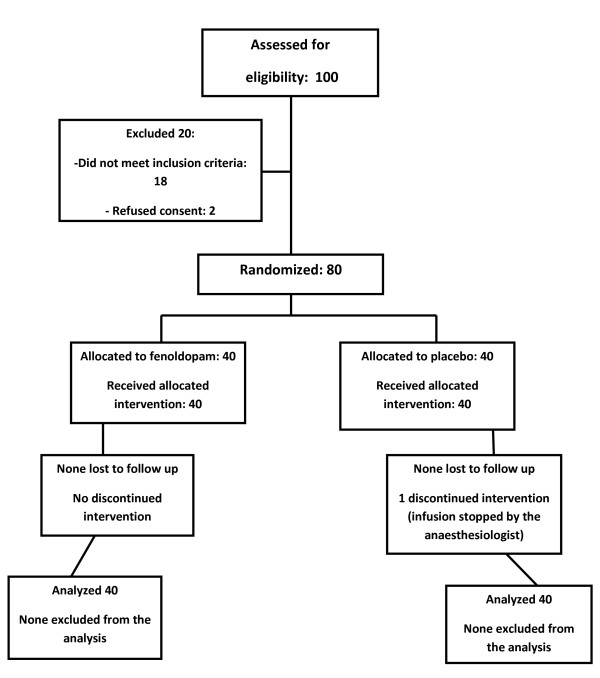
**Flow chart of enrolment phases**.

Figure 1 depicts the flow chart of enrolment phases: in only one case was treatment interrupted by mistake (group P), but data from this patient were analyzed with ITT. Tables 1 and 2 describe baseline characteristics of the two groups that showed similar clinical characteristics.

**Table 1:** Demographic characteristics of enrolled patients and main cardiopulmonary bypass (CPB) features

	Group F	Group P	*P*
Age (days)	145 (156)	139 (130)	0.791
Weight (kg)	5.1 (2.0)	5.0 (2.2)	0.652
BSA (m^2^)	0.5 (0.2)	0.5 (0.3)	0.698
RACHS II score	2.7 (0.6)	2.6 (0.6)	0.366
Surgical procedure (minutes)	390 (84)	401 (82)	0.599
CPB (minutes)	212 (70)	217 (74)	0.905
Crossclamp (minutes)	119 (44)	118 (41)	0.923
Prescribed CPB flow (ml/kg/min)	150 (0)	150 (0)	0.90
Ht during CPB (%)	32 (1.9)	33 (2.1)	0.237
T min during CPB (°)	27.1 (2.5)	27.8 (1.9)	0.418

BSA, body surface area; Ht, hematocrit; RACHS II, risk adjustment for congenital heart surgery; T, central body temperature. Data are expressed as mean (standard deviation).

**Table 2:** Number of neonates and diagnoses of enrolled patients

Diagnoses	Group F	Group P
Number of neonates/total number of patients	13/40	13/40
PA + VSD	2	1
PA + VDS + MAPCAS	2	2
CAVc	5	5
Aortic coarctation and VSD	2	2
ASD-VSD	2	2
Ao supravalvar stenosis	2	2
TOF	12	14
TGA and VSD	2	1
IS-TGA	9	9
TA	2	2
Total	40	40

Ao, aortic; ASD, atrial septal defect; IS, intact septum; MAPCAS, multiple aortopulmonary collaterals; PA, pulmonary atresia; TA, truncus arteriosus; TGA, transposition of the great arteries; TOF, tetralogy of Fallot; VSD, ventricular septal defect.

### Primary end points

**Figure 2:**
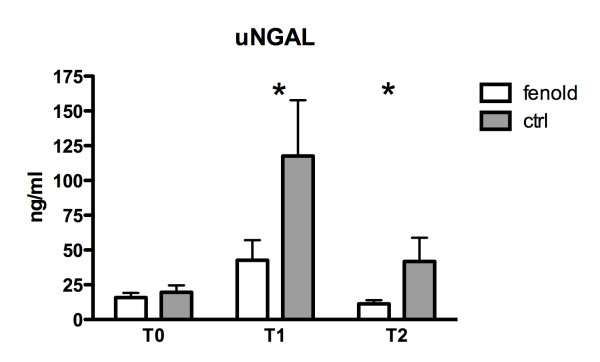
**Urinary neutrophil gelatinase-associated lipocaline (uNGAL) levels**. A significant increase of uNGAL levels from baseline (t0) to ICU admission (t1) and a consequent decrease after 12 hours (t2) was observed in both groups (*P = *0.025). However, uNGAL values were significantly reduced in group F at t1 (*P = *0.02) and t2 (*P = *0.04) compared with group C (*).

**Figure 3:**
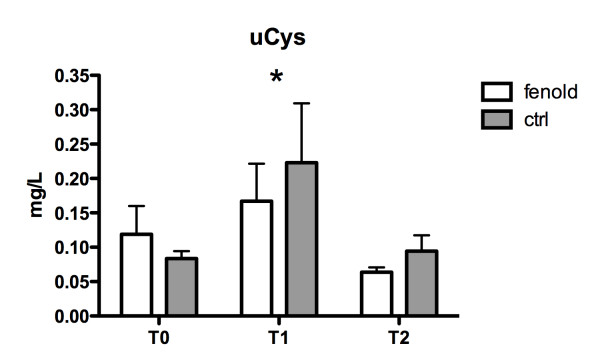
**Urinary cystatin-C (uCysC) levels**. A significant increase of uCysC levels from baseline to ICU admission and a subsequent decrease after 12 hours was observed in both groups (*P = *0.039). UCysC values were significantly reduced at t1 (*P = *0.04) in group F with respect to group C (*).

In both groups, a significant increase of urinary NGAL and CysC levels and a consequent decrease of uCrea was observed from baseline to t1 (*P *= 0.003, 0.03, and 0.0001, respectively), followed by a trend to restoration of normal biomarkers values (Table 3). Considering an average CPB length of 200 minutes and an average total surgery time of 400 minutes in both groups (Table 1), it can be estimated that the t1 NGAL measurement was done in all patients about 150 minutes after CPB stop. However, uNGAL values were significantly reduced at t1 and t2 in group F with respect to group P (Figure 2) (*P *= 0.025); furthermore, if a uNGAL cut-off value of 200 ng/ml was chosen, as previously suggested [35], this value was reached in significantly more children in group P (28%) with respect to group F (5%) (*P *= 0.024; OR, 0.15; CI, 0.03 to 0.75). UCysC values were significantly reduced at t1 in group F with respect to group P (Figure 3) (*P *= 0.039); no differences were observed in uCrea levels between the two groups (Table 3). Plasma biomarkers levels (pNGAL, pCysC, and pCrea) significantly increased from t0 to t1 in both groups (*P = *0.0001 for all biomarkers). PNGAL, pCys, and pCrea values were not significantly different between the two groups (Table 3).

**Table 3:** Renal biomarkers levels in fenoldopam group (F) and placebo group (P) before surgery (t0), at the end of surgical procedure (t1), and 12 hours after intensive care unit admission (t2)

	T0		T1		T2		*P*
	**Group F**	**Group P**	**Group F**	**Group P**	**Group F**	**Group P**	
uNGAL (ng/ml)	37.2 (84.5)	37 (232)	94.2 (240)^a^	140 (249)	20.4 (15.7)^a^	57 (105)	0.025
pNGAL (ng/ml)	62 (33)	66 (31)	88 (38)	96 (40)	93 (45)	99 (41)	0.253
uCys C (mg/l)	0.12 (0.2)	0.08 (0.06)	0.16 (0.3)^a^	0.22 (0.5)	0.06 (0.04)	0.09 (0.13)	0.039
pCys C (mg/l)	1.0 (0.3)	1.1 (0.3)	0.96 (0.28)	1.0 (0.25)	1.19 (0.3)	1.3 (0.3)	0.344
pCrea (mg/dl)	0.51 (0.15)	0.46 (0.16)	0.63 (0.17)	0.64 (0.13)	0.64 (0.2)	0.62 (0.2)	0.114
uCrea (mg/dl)	53.5 (37.4)	49.6 (33.7)	8.5 (5.5)	8.5 (7.6)	26.4 (23.4)	26.4 (23.7)	0.707

Mean (standard deviation) levels of plasma and urinary levels of creatinine (respectively, pCrea and uCrea), neutrophil gelatinase-associated lipocalin (respectively, pNGAL and uNGAL), and cystatine-C (respectively, pCysC and uCysC) are reported. ^a^Significant differences by Bonferroni post test of ANOVA. We found a significant difference between groups of uNGAL levels at t1 (*P = *0.02) and t2 (*P = *0.04) and of uCysC levels at t1 (*P = *0.04). Data are expressed as mean (standard deviation).

### Secondary end points

AKI incidence, as indicated by pRIFLE classification, was 50% in group F and 72% in group P (*P *= 0.08, OR, 0.38; 95% CI, 0.14 to 1.02). AKI patients were classified in 90% of cases in the "Risk" level of severity in both groups, with four "Injury" cases in group F and five in group P. Intra- and postoperative urine output was similar in the two groups (Table 4). Only one patient in group P required postoperative peritoneal dialysis. Average perfusion pressure was 47.16 (4.72) mm Hg in group F and 49.08 (4.61) mm Hg in group P (*P *= 0.048), whereas CPB average effective flow rate was 151 (12.5) ml/kg/min in group F and 146.5 (16) ml/kg/min in group P (*P *= 0.142) (Table 4). ScvO_2_, rSO_2_c, blood lactates, mean indexed SVR, and mean indexed DO_2 _values were not different between groups during the CPB phase (Table 4); however, we observed a significantly higher number of patients with a DO_2 _lower than 272 ml/min/mq (8% vs. 27%; *P *= 0.048; OR, 0.27; 95% CI, 0.06 to 0.9) in the F group, a DO_2 _value recently described as the cut-off level below which risk of AKI occurrence is potentially increased [10]. The number of patients that did not receive any phentolamine and/or furosemide boluses during CPB to target CPB perfusion pressure or urine flow was significantly higher in group F (58%) versus group P (9%) (*P *= 0.0085; OR, 0.22; 95% CI, 0.07 to 0.7). IS did not show significant differences among the two groups either at CPB weaning or in the postoperative phase (Table 4). No side effects were reported in any case of group F. Patients in group F were extubated after 3.8 (3.2) days versus 4.4 (3.7) days for patients in group P (*P *= 0.06). Length of stay was 5.8 (4.8) days in group F versus 7 (5.3) days in group P (*P *= 0.055). All patients survived to hospital discharge.

**Table 4:** Average (standard deviation) levels found

	T0		T1		T2		*P*
	Group F	Group P	Group F	Group P	Group F	Group P	
MAP (mm Hg)	53.1 (8.6)	54.6 (10.7)	62.9 (10.9)	64.6 (14.0)	65.7 (13.0)	62.5 (11.2)	0.99
IS	0 (0)	0 (0)	9.5 (5)	12.7 (9)	8.7 (5)	9.1 (6)	0.374
Lactates (m*M*)	1.0 (0.5)	0.9 (0.5)	3.3 (2.4)	3.8 (2.5)	1.6 (1.0)	1.9 (1.5)	0.433
ScvO_2 _(%)	59.6 (16.6)	59.8 (17.3)	58.1 (15.0)	59.0 (15.3)	61.9 (16.0)	56.7 (14.8)	0.555
rSO_2_c (%)	58.0 (12.1)	58.6 (10.9)	59.8 (15.3)	56.9 (14.7)	64.0 (9.3)	63.5 (11.3)	0.590
Diuresis (ml/kg/h)	5.9 (5.2)	6.9 (6.3)	7.5 (6.7)	6.8 (4.7)	5.0 (2.5)	4.7 (2.8)	0.340
	CPB phase						
	Group F			Group P			
CPB press^a ^(mm Hg)	47.2 (4.7)			49.0 (4.6)			0.048
Effective CPB flow^a ^(ml/kg/min)	151 (12.5)			146.5 (16)			0.142
IDO_2_^a^ (ml O_2_/min/m^2^)	342.4 (50.1)			331.7 (58.7)			0.460
ISVR^a ^(dyn·s·cm^-5^/m^2^)	404.9 (59.8)			423.5 (97.2)			0.502

Levels of mean arterial pressure (MAP), lactates, central venous saturation (ScvO_2_), cerebral near infrared spectroscopy (rSO_2_c), urine output, cardiopulmonary bypass (CPB) pressure, indexed oxygen delivery (IDO_2_), and indexed systemic vascular resistances (ISVR) collected before surgery (t0), at the end of the surgical procedure (t1) and 12 hours after intensive care unit admission (t2). Diuresis at t1 includes intraoperative urine output. ^a^Data collected exclusively during CPB phase. Data are expressed as mean (standard deviation).

## Discussion

Evaluation of organ perfusion and protection during CPB, the gold standard of any CPB procedure, may depend on how carefully organ function is assessed. In light of this, sensitive and specific biomarkers of ischemic kidney injury might be extremely useful in evaluating the efficacy of a CPB strategy on the optimization of renal perfusion. In our study, all enrolled patients experienced NGAL, CysC, (and pCrea) relative increase after CPB with respect to their baseline value, even when such increments did not reach a frankly pathologic cut-off. Nevertheless, this increase was significantly blunted by fenoldopam. Furthermore, fewer patients in group F experienced an increase of uNGAL over 200 ng/ml, recently identified as a reliable cut-off marker of renal function [35]. It is possible that in most cases, such renal impairment would have remained undiagnosed, considering the few patients requiring renal replacement. In a recent large pooled analysis, Haase and co-workers [36] confirmed that a progressive increase in length of hospital stay is present with increasing biomarkers levels, even in patients with normal creatinine. However, analysis of AKI incidence in our population surprisingly showed that 50% of patients in group F and more than 70% in group P were classified in one of the pRIFLE classes.

Adequate systemic DO_2 _during CPB may be one of the most important determinants of "optimal" perfusion, and it has been shown to be an independent risk factor for cardiac surgery-associated AKI [10]. In a cohort of 1,048 coronary artery bypass graft (CABG) patients, Ranucci *et al*. [10] evidenced that an indexed DO_2 _of 272 ml/min/m^2 ^was a critical value for development for postoperative renal dysfunction in a large cohort of adult patients. During our study, only 8% of patients in group F (vs. 27% in group P) reached such a critical level of indexed DO_2_. It must be remarked that it is currently unknown whether such results might be applicable to the pediatric setting. However, during pediatric CPB, management of vascular resistances plays a key role to deliver adequate and homogeneous whole-body DO_2_, so a variety of vasodilator agents have been studied for their effects on tissue perfusion during CPB. Phentolamine is a nonselective competitive alpha1 and alpha2 catecholamine-receptor blocker. It has a half-life of 19 minutes, and it is eliminated mainly by the kidneys. Through postsynaptic receptor inhibition, it has a vasodilating and hypotensive effect that can improve cardiovascular parameters during CPB [17]. More recently, fenoldopam has been used during adult cardiac surgery. The association between its vasodilating proprieties with its specificity on renal (and splanchnic) perfusion makes it a potentially ideal agent during pediatric CPB. Interestingly, in a recent RCT on adult cardiac surgery, patients were randomly assigned to receive the short half-life drug fenoldopam (0.1 μ/kg/min) or placebo [37]. Fenoldopam infusion started at the onset of CPB and was maintained for the first 12 postoperative hours. Patients in the fenoldopam group had higher DO_2 _during CPB, higher pump flow rate, and a significantly lower perfusion pressure, although this parameter was still within the normal range. Blood lactate concentrations during CPB were similar in the two groups. Urine output during and after CPB did not differ between groups, nor did the renal function parameters. A significantly higher rate of AKI was found in the placebo group (10% vs. 0) [37].

In our study, during CPB, we administered 0.1 mg/kg phentolamine boluses in case of high perfusion pressure at full perfusion flow. Similar to the Ranucci *et al*. trial [37], *average *DO_2 _levels, SVR, ScvO_2_, rSO_2_c, and blood lactates suggested adequate perfusion in both groups. However, in the fenoldopam group, CPB average perfusion pressure was significantly lower, and the average effective CPB flow tended to be higher (even higher than the prescribed flow of 150 ml/kg/min). We speculate that a continuous fenoldopam infusion warranted a constant systemic vasodilatation and a better overall perfusion than did repeated phentolamine boluses. This assumption may be supported by the renal biomarker difference, and by the fact that patients in group F required a lower amount of phentolamine and furosemide administration: this effect might be explained, again, by the optimization of renal (and splanchnic) perfusion during CPB. It must be remarked, however, that these speculations derive from an associative analysis, because phentolamine/furosemide administration was not limited by study protocol. This trial had several strengths and pitfalls. This was the first RCT examining the use of high-dose fenoldopam during CPB in pediatric surgery. The trial was stratified by age and included an homogeneous cohort of young babies who might have different CPB physiopathology and systemic vascular resistances from those of older children. The limits of this trial were that it was a single-center study, limiting its external validity; it was not powered for postoperative pediatric AKI; a cost/benefit analysis was not performed; palliative surgery, DHCA procedures, and RACHS 5 and 6 were excluded by protocol from enrolment, whereas in these kinds of patients, the impact of perfusion optimization on organ protection would have been potentially magnified; finally, only indices of renal function were strictly monitored and eventually considered as surrogates of splanchnic perfusion. However, it must be remarked that this was an initial experience, and the potential feasibility of high-dose fenoldopam during pediatric CPB has not been evaluated before; therefore, a sensitive and specific biomarker of renal ischemic injury was targeted as the primary objective instead of cardiac surgery-associated AKI or other organs dysfunction, due to the specific fenoldopam action on renal perfusion: this primary outcome allowed us to reduce sample size and costs of the trial; furthermore, the administration of the drug was limited to a "safer" cohort of CHD, at lower risk of perioperative morbidity and unstable hemodynamics. After such results, the extended application of high-dose fenoldopam to patients with univentricular anatomy and higher RACHS score is currently under evaluation in our institution.

## Conclusions

In conclusion, high-dose fenoldopam during CPB in pediatric cardiac surgery appeared to be safe and potentially nephroprotective. The routine prophylactic use of this agent during CPB cannot be recommended on the basis of this trial, especially before an accurate cost/benefit analysis is performed; however, our data encourage further research in this field.

## Key messages

• Cardiopulmonary bypass during pediatric cardiac surgery induces a significant increase of urinary and plasmatic NGAL and CysC levels from baseline to ICU admission followed by restoration of normal values after 12 hours.

• Urinary NGAL and CysC increase is significantly reduced at the end of surgery and 12 hours after ICU admission by the infusion of 1 μg/kg/min of fenoldopam.

• Fenoldopam infusion may improve systemic vasodilation and renal perfusion during CPB: compared with controls, a significant reduction in furosemide and phentolamine requirement during CPB was observed in patients receiving fenoldopam.

• AKI incidence, as indicated by pRIFLE classification after pediatric cardiac surgery, is high. According to our data, it was 50% in patients receiving fenoldopam and 72% in controls.

## Abbreviations

AKI: acute kidney injury; CHD: congenital heart disease; CPB: cardiopulmonary bypass; Crea: creatinine; CysC: cystatin C; DHCA: deep hypothermic circulatory arrest; DO_2_: oxygen delivery; ICU: intensive care unit; IS: inotropic score; MAP: mean arterial pressure; NGAL: neutrophil gelatinase-associated lipocaline; pRIFLE: pediatric RIFLE (Risk: Injury: Failure: Loss of function: End-stage kidney disease); rSO_2_c: cerebral near-infrared spectroscopy; ScvO_2_: superior vena cava oxygen saturation; SVR: systemic vascular resistance.

## Competing interests

The authors declare that they have no competing interests.

## Authors' contributions

ZR contributed to the idea and the design of the study, was responsible for acquisition of patient data, collected and analyzed the data, and wrote the manuscript. RL performed analyses, contributed to the drafts of the manuscript, and gave final approval to the present manuscript. IF, CG, SM, and LDC contributed to data collection, assisted in patient recruitment, and gave final approval to the present manuscript. MM contributed to study conception, supervised analyses performance, and gave final approval to the present manuscript. SP participated in study conception, design, and coordination, helped in manuscript revision, and gave final approval to the present manuscript. PC helped to draft the manuscript, participated in interpretation of the data, supervised the final manuscript version, and gave final approval to the present manuscript.
